# The effectiveness of virtual reality training on knowledge, skills and attitudes of health care professionals and students in assessing and treating mental health disorders: a systematic review

**DOI:** 10.1186/s12909-024-05423-0

**Published:** 2024-05-01

**Authors:** Cathrine W. Steen, Kerstin Söderström, Bjørn Stensrud, Inger Beate Nylund, Johan Siqveland

**Affiliations:** 1 Mental Health Department, Innlandet Hospital Trust, P.B 104, Brumunddal, 2381 Norway; 2 Inland Norway University of Applied Sciences, P.B. 400, Elverum, 2418 Norway; 3 Norwegian National Advisory Unit On Concurrent Substance Abuse and Mental Health Disorders, Innlandet Hospital Trust, P.B 104, Brumunddal, 2381 Norway; 4 Akershus University Hospital, P.B 1000, Lørenskog, 1478 Norway; 5 National Centre for Suicide Research and Prevention, Oslo, 0372 Norway

**Keywords:** Health care professionals, Health care students, Virtual reality, Training, Mental health, Clinical skills, Systematic review

## Abstract

**Background:**

Virtual reality (VR) training can enhance health professionals’ learning. However, there are ambiguous findings on the effectiveness of VR as an educational tool in mental health. We therefore reviewed the existing literature on the effectiveness of VR training on health professionals’ knowledge, skills, and attitudes in assessing and treating patients with mental health disorders.

**Methods:**

We searched MEDLINE, PsycINFO (via Ovid), the Cochrane Library, ERIC, CINAHL (on EBSCOhost), Web of Science Core Collection, and the Scopus database for studies published from January 1985 to July 2023. We included all studies evaluating the effect of VR training interventions on attitudes, knowledge, and skills pertinent to the assessment and treatment of mental health disorders and published in English or Scandinavian languages. The quality of the evidence in randomized controlled trials was assessed with the Cochrane Risk of Bias Tool 2.0. For non-randomized studies, we assessed the quality of the studies with the ROBINS-I tool.

**Results:**

Of 4170 unique records identified, eight studies were eligible. The four randomized controlled trials were assessed as having some concern or a high risk of overall bias. The four non-randomized studies were assessed as having a moderate to serious overall risk of bias. Of the eight included studies, four used a virtual standardized patient design to simulate training situations, two studies used interactive patient scenario training designs, while two studies used a virtual patient game design. The results suggest that VR training interventions can promote knowledge and skills acquisition.

**Conclusions:**

The findings indicate that VR interventions can effectively train health care personnel to acquire knowledge and skills in the assessment and treatment of mental health disorders. However, study heterogeneity, prevalence of small sample sizes, and many studies with a high or serious risk of bias suggest an uncertain evidence base. Future research on the effectiveness of VR training should include assessment of immersive VR training designs and a focus on more robust studies with larger sample sizes.

**Trial registration:**

This review was pre-registered in the Open Science Framework register with the ID-number Z8EDK.

**Supplementary Information:**

The online version contains supplementary material available at 10.1186/s12909-024-05423-0.

## Background

A robustly trained health care workforce is pivotal to forging a resilient health care system [1], and there is an urgent need to develop innovative methods and emerging technologies for health care workforce education [2]. Virtual reality technology designs for clinical training have emerged as a promising avenue for increasing the competence of health care professionals, reflecting their potential to provide effective training [3].

Virtual reality (VR) is a dynamic and diverse field, and can be described as a computer-generated environment that simulates sensory experiences, where user interactions play a role in shaping the course of events within that environment [4]. When optimally designed, VR gives users the feeling that they are physically within this simulated space, unlocking its potential as a dynamic and immersive learning tool [5]. The cornerstone of the allure of VR is its capacity for creating artificial settings via sensory deceptions, encapsulated by the term ‘immersion’. Immersion conveys the sensation of being deeply engrossed or enveloped in an alternate world, akin to absorption in a video game. Some VR systems will be more immersive than others, based on the technology used to influence the senses. However, the degree of immersion does not necessarily determine the user’s level of engagement with the application [6].

A common approach to categorizing VR systems is based on the design of the technology used, allowing them to be classified into: 1) non-immersive desktop systems, where users experience virtual environments through a computer screen, 2) immersive CAVE systems with large projected images and motion trackers to adjust the image to the user, and 3) fully immersive head-mounted display systems that involve users wearing a headset that fully covers their eyes and ears, thus entirely immersing them in the virtual environment [7]. Advances in VR technology have enabled a wide range of VR experiences. The possibility for health care professionals to repeatedly practice clinical skills with virtual patients in a risk-free environment offers an invaluable learning platform for health care education.

The impact of VR training on health care professionals’ learning has predominantly been researched in terms of the enhancement of technical surgical abilities. This includes refining procedural planning, familiarizing oneself with medical instruments, and practicing psychomotor skills such as dexterity, accuracy, and speed [8, 9]. In contrast, the exploration of VR training in fostering non-technical or ‘soft’ skills, such as communication and teamwork, appears to be less prevalent [10]. A recent systematic review evaluates the outcomes of VR training in non-technical skills across various medical specialties [11], focusing on vital cognitive abilities (e.g., situation awareness, decision-making) and interprofessional social competencies (e.g., teamwork, conflict resolution, leadership). These skills are pivotal in promoting collaboration among colleagues and ensuring a safe health care environment. At the same time, they are not sufficiently comprehensive for encounters with patients with mental health disorders.

For health care professionals providing care to patients with mental health disorders, acquiring specific skills, knowledge, and empathic attitudes is of utmost importance. Many individuals experiencing mental health challenges may find it difficult to communicate their thoughts and feelings, and it is therefore essential for health care providers to cultivate an environment where patients feel safe and encouraged to share feelings and thoughts. Beyond fostering trust, health care professionals must also possess in-depth knowledge about the nature and treatment of various mental health disorders. Moreover, they must actively practice and internalize the skills necessary to translate their knowledge into clinical practice. While the conventional approach to training mental health clinical skills has been through simulation or role-playing with peers under expert supervision and practicing with real patients, the emergence of VR applications presents a compelling alternative. This technology promises a potentially transformative way to train mental health professionals. Our review identifies specific outcomes in knowledge, skills, and attitudes, covering areas from theoretical understanding to practical application and patient interaction. By focusing on these measurable concepts, which are in line with current healthcare education guidelines [12], we aim to contribute to the knowledge base and provide a detailed analysis of the complexities in mental health care training. This approach is designed to highlight the VR training’s practical relevance alongside its contribution to academic discourse.

A recent systematic review evaluated the effects of virtual patient (VP) interventions on knowledge, skills, and attitudes in undergraduate psychiatry education [13]. This review’s scope is limited to assessing VP interventions and does not cover other types of VR training interventions. Furthermore, it adopts a classification of VP different from our review, rendering their findings and conclusions not directly comparable to ours.

To the best of our knowledge, no systematic review has assessed and summarized the effectiveness of VR training interventions for health professionals in the assessment and treatment of mental health disorders. This systematic review addresses the gap by exploring the effectiveness of virtual reality in the training of knowledge, skills, and attitudes health professionals need to master in the assessment and treatment of mental health disorders.

## Methods

This systematic review follows the guidelines of Preferred Reporting Items for Systematic Reviews and Meta-Analysis [14]. The protocol of the systematic review was registered in the Open Science Framework register with the registration ID Z8EDK.

We included randomized controlled trials, cohort studies, and pretest–posttest studies, which met the following criteria: a) a population of health care professionals or health care professional students, b) assessed the effectiveness of a VR application in assessing and treating mental health disorders, and c) reported changes in knowledge, skills, or attitudes. We excluded studies evaluating VR interventions not designed for training in assessing and treating mental health disorders (e.g., training of surgical skills), studies evaluating VR training from the first-person perspective, studies that used VR interventions for non-educational purposes and studies where VR interventions trained patients with mental health problems (e.g., social skills training). We also excluded studies not published in English or Scandinavian languages.

### Search strategy

The literature search reporting was guided by relevant items in PRISMA-S [15]. In collaboration with a senior academic librarian (IBN), we developed the search strategy for the systematic review. Inspired by the ‘pearl harvesting’ information retrieval approach [16], we anticipated a broad spectrum of terms related to our interdisciplinary query. Recognizing that various terminologies could encapsulate our central ideas, we harvested an array of terms for each of the four elements ‘health care professionals and health care students’, ‘VR’, ‘training’, and ‘mental health’. The pearl harvesting framework [16] consists of four steps which we followed with some minor adaptions. Step 1: We searched for and sampled a set of relevant research articles, a book chapter, and literature reviews. Step 2: The librarian scrutinized titles, abstracts, and author keywords, as well as subject headings used in databases, and collected relevant terms. Step 3: The librarian refined the lists of terms. Step 4: The review group, in collaboration with a VR consultant from KildeGruppen AS (a Norwegian media company), validated the refined lists of terms to ensure they included all relevant VR search terms. This process for the element VR resulted in the inclusion of search terms such as ‘3D simulated environment’, ‘second life simulation’, ‘virtual patient’, and ‘virtual world’. We were given a peer review of the search strategy by an academic librarian at Inland Norway University of Applied Sciences.

**Fig. 1:**
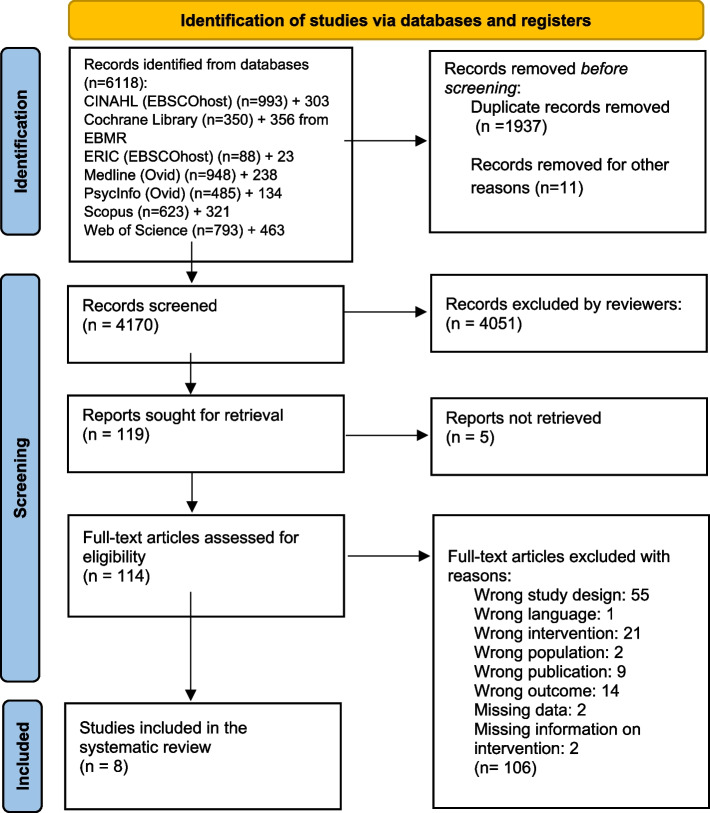
PRISMA flow chart of the records and study selection process

In June and July 2021, we performed comprehensive searches for publications dating from January 1985 to the present. This period for the inclusion of studies was chosen since VR systems designed for training in health care first emerged in the early 1990s. The searches were carried out in seven databases: MEDLINE and PsycInfo (on Ovid), ERIC and CINAHL (on EBSCOhost), the Cochrane Library, Web of Science Core Collection, and Scopus. Detailed search strategies from each database are available for public access at DataverseNO [17]. On July 2, 2021, a search in CINAHL yielded 993 hits. However, when attempting to transfer these records to EndNote using the ‘Folder View’—a feature designed for organizing and managing selected records before export—only 982 records were successfully transferred. This discrepancy indicates that 11 records could not be transferred through Folder View, for reasons not specified. The process was repeated twice, consistently yielding the same discrepancy. The missing 11 records pose a risk of failing to capture relevant studies in the initial search. In July 2023, to make sure that we included the latest publications, we updated our initial searches, focusing on entries since January 1, 2021. This ensured that we did not miss any new references recently added to these databases. Due to a lack of access to the Cochrane Library in July 2023, we used EBMR (Evidence Based Medicine Reviews) on the Ovid platform instead, including the databases Cochrane Central Register of Controlled Trials, Cochrane Database of Systematic Reviews, and Cochrane Clinical Answers. All references were exported to Endnote and duplicates were removed. The number of records from each database can be observed in the PRISMA diagram [14], Fig. 1.

### Study selection and data collection

Two reviewers (JS, CWS) independently assessed the titles and abstracts of studies retrieved from the literature search based on the eligibility criteria. We employed the Rayyan website for the screening process [18]. The same reviewers (JS, CWS) assessed the full-text articles selected after the initial screening. Articles meeting the eligibility criteria were incorporated into the review. Any disagreements were resolved through discussion.

Data extracted from the studies by the first author (CWS) and cross-checked by another reviewer (JS) included: authors of the study, publication year, country, study design, participant details (education, setting), interventions (VR system, class label), comparison types, outcomes, and main findings. This data is summarized in Table 1 and Additional file 1. In the process of reviewing the VR interventions utilized within the included studies, we sought expertise from advisers associated with VRINN, a Norwegian immersive learning cluster, and SIMInnlandet, a center dedicated to simulation in mental health care at Innlandet Hospital Trust. This collaboration ensured a thorough examination and accurate categorization of the VR technologies applied. Furthermore, the classification of the learning designs employed in the VP interventions was conducted under the guidance of an experienced VP scholar at Paracelcus Medical University in Salzburg.

**Table 1:** Characteristics of the included studies: Randomized controlled trials (RCTs) and non-randomized studies

Author, year, country, study design	Participants	Topic	VR training intervention, VR system	Type of VR training intervention Duration	Comparison	Outcome, outcome measurement
Albright et al., 2018, USA, RCT [19]	Primary health care professionals: nurses, nurse practitioners and doctors *n* = 227	Mental health and substance use disorders	Non-immersive desktop system	VP game/virtual world One session, 60–90 min	Control group: no intervention	Knowledge and skills A knowledge and skills scale: Self-reported knowledge and skills were evaluated using a composite score from six Gatekeeper Behavior Scale items, assessed before and after the simulation.
Fleming et al., 2009, USA, RCT [20]	Health professionals and health professional students *n* = 102	Substance use	Non-immersive desktop system	Virtual standardized patient At least ten sessions during a three-month study period, case durations not specified	Control group: no intervention	Skills Standardized patient with checklist: Eighty-five points was allocated to 17 specific criteria, with five points for each. Fifteen points were used to rate overall performance, combined into a total score per scenario.
Foster et al., 2015, USA, RCT [21]	Second-year medical college students *n* = 67	Suicide risk assessment	Non-immersive desktop system	Virtual standardized patient One session, duration not specified	Control group: watched a video of a physician’s interview with a patient	Skills Standardized patient with checklist: A 19-item yes/no checklist, with the primary outcome being how often students asked questions about suicide risk.
Satter, 2012, USA, RCT [22]	Family physicians *n* = 30	Major depressive disorder, posttraumatic stress disorder	Non-immersive desktop system	Virtual standardized patient One session, 10–40 min	Control group: Text based simulation	Skills Diagnostic form: Participants' responses for each diagnostic case were coded for accuracy on a 1–7 scale.
Hitchcock et al., 2019, USA, single group pretest–posttest design [23]	Social work and nursing students *n* = 100	Substance use	Non-immersive desktop system	Virtual standardized patient One session, duration not specified	No comparison	Attitudes, perceived competence Online pretest- posttest survey with 7 questions from the Brief Substance Abuse Attitudes Scale, and 17 questions assessing perceived competence.
Liu, 2021, USA, prospective cohort study [24]	Undergraduate nursing students *n* = 299	Depression, schizophrenia	Non-immersive desktop system	VP game/virtual world Number of virtual simulations not specified; case durations not specified	Comparison: No intervention	Knowledge Recognition of diagnosis: Students were asked to identify the correct diagnosis for two case vignettes of depression and schizophrenia.
Matsumara et al., 2018, Japan, controlled before and after study [25]	Medical students in psychiatric clinical training *n* = 79	Dementia	Non-immersive desktop system	Interactive patient scenario design One session, 45 min	Comparison: No intervention	Knowledge Preintervention and postintervention tests: Students responded by inputting 27 keywords, receiving one point for each correct entry, with a potential score range of 0 to 27.
Pantziaras et al., 2015, Sweden, single group pretest- posttest design [26]	Resident psychiatrists *n* = 32	Posttraumatic stress disorder	Non-immersive desktop system	Interactive patient scenario design One session, 25–110 min	No comparison	Knowledge Knowledge test: Eleven multiple-choice questions were administered, with a maximum score of 11, as each correct answer was awarded one point.

### Data analysis

We initially intended to perform a meta-analysis with knowledge, skills, and attitudes as primary outcomes, planning separate analyses for each. However, due to significant heterogeneity observed among the included studies, it was not feasible to carry out a meta-analysis. Consequently, we opted for a narrative synthesis based on these pre-determined outcomes of knowledge, skills, and attitudes. This approach allowed for an analysis of the relationships both within and between the studies. The effect sizes were calculated using a web-based effect size calculator [27]. We have interpreted effect sizes based on commonly used descriptions for Cohen’s d: small = 0.2, moderate = 0.5, and large = 0.8, and for Cramer’s V: small = 0.10, medium = 0.30, and large = 0.50.

### Risk of bias assessment

JS and CWS independently evaluated the risk of bias for all studies using two distinct assessment tools. We used the Cochrane risk of bias tool RoB 2 [28] to assess the risk of bias in the RCTs. With the RoB 2 tool, the bias was assessed as high, some concerns or low for five domains: randomization process, deviations from the intended interventions, missing outcome data, measurement of the outcome, and selection of the reported result [28].

We used the Risk Of Bias In Non-randomized Studies of Interventions (ROBINS-I) tool [29] to assess the risk of bias in the cohort and single-group studies. By using ROBINS-I for the non-randomized trials, the risk of bias was assessed using the categories low, moderate, serious, critical or no information for seven domains: confounding, selection of participants, classification of interventions, deviations from intended interventions, missing data, measurement of outcomes, and selection of the reported result [29].

## Results

We included eight studies in the review (Fig. 1). An overview of the included studies is presented in detail in Table 1.

Four studies were RCTs [19–22], two were single group pretest–posttest studies [23, 26], one was a controlled before and after study [25], and one was a cohort study [24]. The studies included health professionals from diverse educational backgrounds, including some from mental health and medical services, as well as students in medicine, social work, and nursing. All studies, published from 2009 to 2021, utilized non-immersive VR desktop system interventions featuring various forms of VP designs. Based on an updated classification of VP interventions by Kononowicz et al. [30] developed from a model proposed by Talbot et al. [31], we have described the characteristics of the interventions in Table 1. Four of the studies utilized a virtual standardized patient (VSP) intervention [20–23], a conversational agent that simulates clinical presentations for training purposes. Two studies employed an interactive patient scenario (IPS) design [25, 26], an approach that primarily uses text-based multimedia, enhanced with images and case histories through text or voice narratives, to simulate clinical scenarios. Lastly, two studies used a virtual patient game (VP game) intervention [19, 24]. These interventions feature training scenarios using 3D avatars, specifically designed to improve clinical reasoning and team training skills. It should be noted that the interventions classified as VSPs in this review, being a few years old, do not encompass artificial intelligence (AI) as we interpret it today. However, since the interventions include some kind of algorithm that provides answers to questions, we consider them as conversational agents, and therefore as VSPs. As the eight included studies varied significantly in terms of design, interventions, and outcome measures, we could not incorporate them into a meta-analysis.

### Risk of bias assessment

**Fig. 2:**
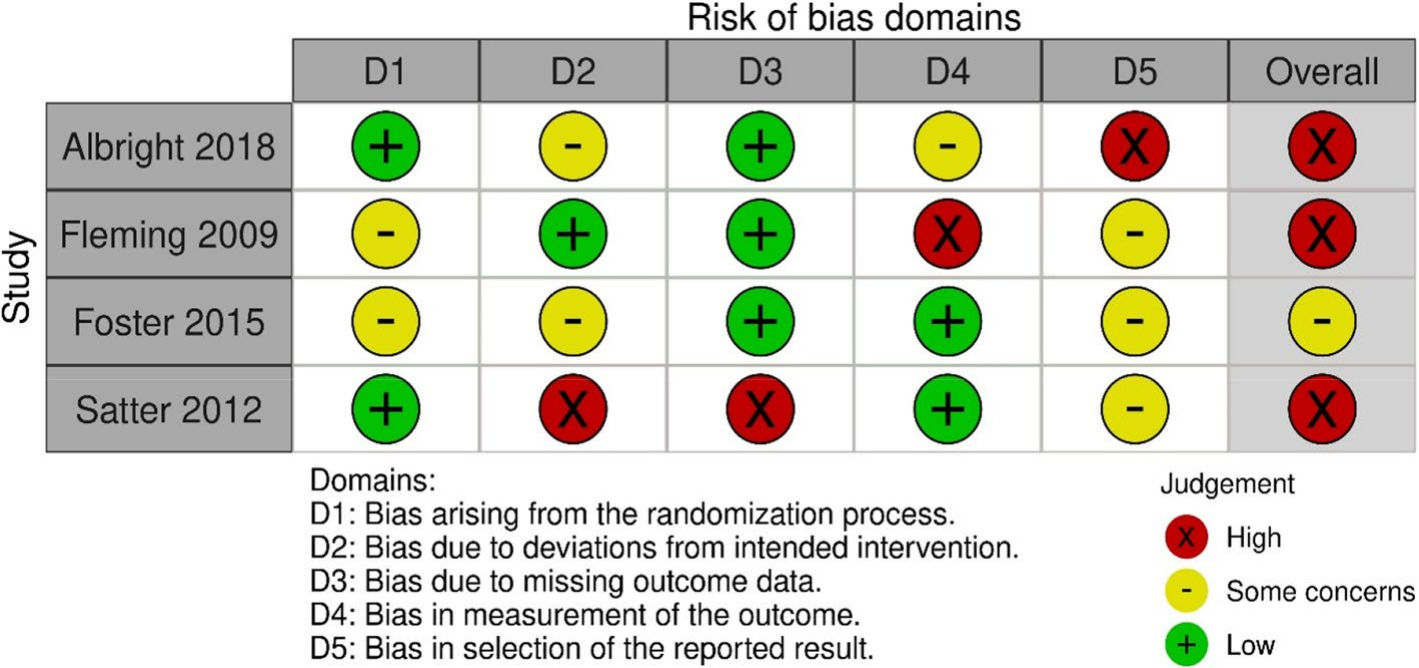
Risk of bias summary: review authors assessments of each risk of bias item in the included RCT studies

The overall risk of bias for the four RCTs was high [19, 20, 22] or of some concern [21] (Fig. 2). They were all assessed as low or of some concern in the domains of randomization. Three studies were assessed with a high risk of bias in one [19, 20] or two domains [22]; one study had a high risk of bias in the domain of selection of the reported result [19], one in the domain of measurement of outcome [20], and one in the domains of deviation from the intended interventions and missing outcome data [22]. One study was not assessed as having a high risk of bias in any domain [21].

**Fig. 3:**
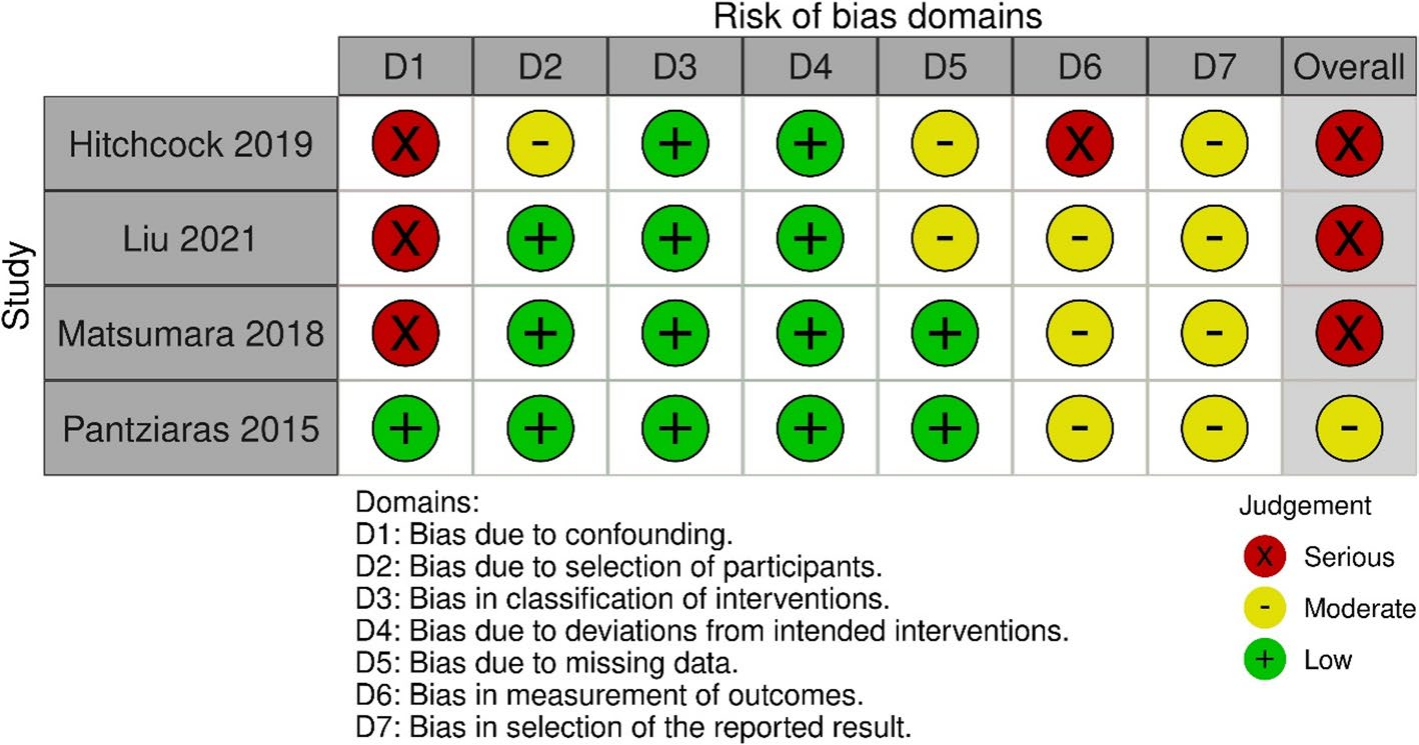
Risk of bias summary: review authors assessments of each risk of bias item in the included non-randomized studies

For the four non-randomized studies, the overall risk of bias was judged to be moderate [26] or serious [23–25] (Fig. 3). One study had a serious risk of bias in two domains: confounding and measurement of outcomes [23]. Two studies had a serious risk of bias in one domain, namely confounding [24, 25], while one study was judged not to have a serious risk of bias in any domain [26].

### Outcomes

#### Knowledge

Three studies investigated the impact of virtual reality training on mental health knowledge [24–26]. One study with 32 resident psychiatrists in a single group pretest–posttest design assessed the effect of a VR training intervention on knowledge of posttraumatic stress disorder (PTSD) symptomatology, clinical management, and communication skills [26]. The intervention consisted of an IPS. The assessment of the outcome was conducted using a knowledge test with 11 multiple-choice questions and was administered before and after the intervention. This study reported a significant improvement on the knowledge test after the VR training intervention.

The second study examined the effect of a VR training intervention on knowledge of dementia [25], employing a controlled before and after design. Seventy-nine medical students in clinical training were divided into two groups, following a traditional learning program. The experimental group received an IPS intervention. The outcome was evaluated with a knowledge test administered before and after the intervention with significantly higher posttest scores in the experimental group than in the control group, with a moderate effects size observed between the groups.

A third study evaluated the effect of a VR training intervention on 299 undergraduate nursing students’ diagnostic recognition of depression and schizophrenia (classified as knowledge) [24]. In a prospective cohort design, the VR intervention was the only difference in the mental health related educational content provided to the two cohorts, and consisted of a VP game design, developed to simulate training situations with virtual patient case scenarios, including depression and schizophrenia. The outcome was assessed by determining the accuracy of diagnoses made after reviewing case vignettes of depression and schizophrenia. The study found no statistically significant effect of VR training on diagnostic accuracy between the simulation and the non-simulation cohort.

Summary: All three studies assessing the effect of a VR intervention on knowledge were non-randomized studies with different study designs using different outcome measures. Two studies used an IPS design, while one study used a VP game design. Two of the studies found a significant effect of VR training on knowledge. Of these, one study had a moderate overall risk of bias [26], while the other was assessed as having a serious overall risk of bias [25]. The third study, which did not find any effect of the virtual reality intervention on knowledge, was assessed to have a serious risk of bias [24].

#### Skills

Three RCTs assessed the effectiveness of VR training on skills [20–22]. One of them evaluated the effect of VR training on clinical skills in alcohol screening and intervention [20]. In this study, 102 health care professionals were randomly allocated to either a group receiving no training or a group receiving a VSP intervention. To evaluate the outcome, three standardized patients rated each participant using a checklist based on clinical criteria. The VSP intervention group demonstrated significantly improved posttest skills in alcohol screening and brief intervention compared to the control group, with moderate and small effect sizes, respectively.

Another RCT, including 67 medical college students, evaluated the effect of VR training on clinical skills by comparing the frequency of questions asked about suicide in a VSP intervention group and a video module group [21]. The assessment of the outcome was a psychiatric interview with a standardized patient. The primary outcome was the frequency with which the students asked the standardized patient five questions about suicide risk. Minimal to small effect sizes were noted in favor of the VSP intervention, though they did not achieve statistical significance for any outcomes.

One posttest only RCT evaluated the effect of three training programs on skills in detecting and diagnosing major depressive disorder and posttraumatic stress disorder (PTSD) [22]. The study included 30 family physicians, and featured interventions that consisted of two different VSPs designed to simulate training situations, and one text-based program. A diagnostic form filled in by the participants after the intervention was used to assess the outcome. The results revealed a significant effect on diagnostic accuracy for major depressive disorder for both groups receiving VR training, compared to the text-based program, with large effect sizes observed. For PTSD, the intervention using a fixed avatar significantly improved diagnostic accuracy with a large effect size, whereas the intervention with a choice avatar demonstrated a moderate to large effect size compared to the text-based program.

Summary: Three RCTs assessed the effectiveness of VR training on clinical skills [20–22], all of which used a VSP design. To evaluate the effect of training, two of the studies utilized standardized patients with checklists. The third study measured the effect on skills using a diagnostic form completed by the participants. Two of the studies found a significant effect on skills [20, 22], both were assessed to have a high risk of bias. The third study, which did not find any effect of VR training on skills, had some concern for risk of bias [21].

#### Knowledge and skills

One RCT study with 227 health care professionals assessed knowledge and skills as a combined outcome compared to a waitlist control group, using a self-report survey before and after the VR training [19]. The training intervention was a VP game designed to practice knowledge and skills related to mental health and substance abuse disorders. To assess effect of the training, participants completed a self-report scale measuring perceived knowledge and skills. Changes between presimulation and postsimulation scores were reported only for the within treatment group (*n* = 117), where the composite postsimulation score was significantly higher than the presimulation score, with a large effect size observed. The study was judged to have a high risk of bias in the domain of selection of the reported result.

#### Attitudes

One single group pretest–posttest study with 100 social work and nursing students assessed the effect of VSP training on attitudes towards individuals with substance abuse disorders [23]. To assess the effect of the training, participants completed an online pretest and posttest survey including questions from a substance abuse attitudes survey. This study found no significant effect of VR training on attitudes and was assessed as having a serious risk of bias.

#### Perceived competence

The same single group pretest–posttest study also assessed the effect of a VSP training intervention on perceived competence in screening, brief intervention, and referral to treatment in encounters with patients with substance abuse disorders [23]. A commonly accepted definition of competence is that it comprises integrated components of knowledge, skills, and attitudes that enable the successful execution of a professional task [32]. To assess the effect of the training, participants completed an online pretest and posttest survey including questions on perceived competence. The study findings demonstrated a significant increase in perceived competence following the VSP intervention. The risk of bias in this study was judged as serious.

## Discussion

This systematic review aimed to investigate the effectiveness of VR training on knowledge, skills, and attitudes that health professionals need to master in the assessment and treatment of mental health disorders. A narrative synthesis of eight included studies identified VR training interventions that varied in design and educational content. Although mixed results emerged, most studies reported improvements in knowledge and skills after VR training.

We found that all interventions utilized some type of VP design, predominantly VSP interventions. Although our review includes a limited number of studies, it is noteworthy that the distribution of interventions contrasts with a literature review on the use of ‘virtual patient’ in health care education from 2015 [30], which identified IPS as the most frequent intervention. This variation may stem from our review’s focus on the mental health field, suggesting a different intervention need and distribution than that observed in general medical education. A fundamental aspect of mental health education involves training skills needed for interpersonal communication, clinical interviews, and symptom assessment, which makes VSPs particularly appropriate. While VP games may be suitable for clinical reasoning in medical fields, offering the opportunity to perform technical medical procedures in a virtual environment, these designs may present some limitations for skills training in mental health education. Notably, avatars in a VP game do not comprehend natural language and are incapable of engaging in conversations. Therefore, the continued advancement of conversational agents like VSPs is particularly compelling and considered by scholars to hold the greatest potential for clinical skills training in mental health education [3]. VSPs, equipped with AI dialogue capabilities, are particularly valuable for repetitive practice in key skills such as interviewing and counseling [31], which are crucial in the assessment and treatment of mental health disorders. VSPs could also be a valuable tool for the implementation of training methods in mental health education, such as deliberate practice, a method that has gained attention in psychotherapy training in recent years [33] for its effectiveness in refining specific performance areas through consistent repetition [34]. Within this evolving landscape, AI system-based large language models (LLMs) like ChatGPT stand out as a promising innovation. Developed from extensive datasets that include billions of words from a variety of sources, these models possess the ability to generate and understand text in a manner akin to human interaction [35]. The integration of LLMs into educational contexts shows promise, yet careful consideration and thorough evaluation of their limitations are essential [36]. One concern regarding LLMs is the possibility of generating inaccurate information, which represents a challenge in healthcare education where precision is crucial [37]. Furthermore, the use of generative AI raises ethical questions, notably because of potential biases in the training datasets, including content from books and the internet that may not have been verified, thereby risking the perpetuation of these biases [38]. Developing strategies to mitigate these challenges is imperative, ensuring LLMs are utilized safely in healthcare education.

All interventions in our review were based on non-immersive desktop VR systems, which is somewhat surprising considering the growing body of literature highlighting the impact of immersive VR technology in education, as exemplified by reviews such as that of Radianti et al. [39]. Furthermore, given the recent accessibility of affordable, high-quality head-mounted displays, this observation is noteworthy. Research has indicated that immersive learning based on head-mounted displays generally yields better learning outcomes than non-immersive approaches [40], making it an interesting research area in mental health care training and education. Studies using immersive interventions were excluded in the present review because of methodological concerns, paralleling findings described in a systematic review on immersive VR in education [41], suggesting the potential early stage of research within this field. Moreover, the integration of immersive VR technology into mental health care education may encounter challenges associated with complex ethical and regulatory frameworks, including data privacy concerns exemplified by the Oculus VR headset-Facebook integration, which could restrict the implementation of this technology in healthcare setting. Prioritizing specific training methodologies for enhancing skills may also affect the utilization of immersive VR in mental health education. For example, integrating interactive VSPs into a fully immersive VR environment remains a costly endeavor, potentially limiting the widespread adoption of immersive VR in mental health care. Meanwhile, the use of 360-degree videos in immersive VR environments for training purposes [42] can be realized with a significantly lower budget. Immersive VR offers promising opportunities for innovative training, but realizing its full potential in mental health care education requires broader research validation and the resolution of existing obstacles.

This review bears some resemblance to the systematic review by Jensen et al. on virtual patients in undergraduate psychiatry education [13] from 2024, which found that virtual patients improved learning outcomes compared to traditional methods. However, these authors’ expansion of the commonly used definition of virtual patient makes their results difficult to compare with the findings in the present review. A recognized challenge in understanding VR application in health care training arises from the literature on VR training for health care personnel, where ‘virtual patient’ is a term broadly used to describe a diverse range of VR interventions, which vary significantly in technology and educational design [3, 30]. For instance, reviews might group different interventions using various VR systems and designs under a single label (virtual patient), or primary studies may use misleading or inadequately defined classifications for the virtual patient interventions evaluated. Clarifying the similarities and differences among these interventions is vital to inform development and enhance communication and understanding in educational contexts [43].

### Strengths and limitations

To the best of our knowledge, this is the first systematic review to evaluate the effectiveness of VR training on knowledge, skills, and attitudes in health care professionals and students in assessing and treating mental health disorders. This review therefore provides valuable insights into the use of VR technology in training and education for mental health care. Another strength of this review is the comprehensive search strategy developed by a senior academic librarian at Inland Norway University of Applied Sciences (HINN) and the authors in collaboration with an adviser from KildeGruppen AS (a Norwegian media company). The search strategy was peer-reviewed by an academic librarian at HINN. Advisers from VRINN (an immersive learning cluster in Norway) and SIMInnlandet (a center for simulation in mental health care at Innlandet Hospital Trust) provided assistance in reviewing the VR systems of the studies, while the classification of the learning designs was conducted under the guidance of a VP scholar. This systematic review relies on an established and recognized classification of VR interventions for training health care personnel and may enhance understanding of the effectiveness of VR interventions designed for the training of mental health care personnel.

This review has some limitations. As we aimed to measure the effect of the VR intervention alone and not the effect of a blended training design, the selection of included studies was limited. Studies not covered in this review might have offered different insights. Given the understanding that blended learning designs, where technology is combined with other forms of learning, have significant positive effects on learning outcomes [44], we were unable to evaluate interventions that may be more effective in clinical settings. Further, by limiting the outcomes to knowledge, skills, and attitudes, we might have missed insights into other outcomes that are pivotal to competence acquisition.

Limitations in many of the included studies necessitate cautious interpretation of the review’s findings. Small sample sizes and weak designs in several studies, coupled with the use of non-validated outcome measures in some studies, diminish the robustness of the findings. Furthermore, the risk of bias assessment in this review indicates a predominantly high or serious risk of bias across most of the studies, regardless of their design. In addition, the heterogeneity of the studies in terms of study design, interventions, and outcome measures prevented us from conducting a meta-analysis.

### Further research

Future research on the effectiveness of VR training for specific learning outcomes in assessing and treating mental health disorders should encompass more rigorous experimental studies with larger sample sizes. These studies should include verifiable descriptions of the VR interventions and employ validated tools to measure outcomes. Moreover, considering that much professional learning involves interactive and reflective practice, research on VR training would probably be enhanced by developing more in-depth study designs that evaluate not only the immediate learning outcomes of VR training but also the broader learning processes associated with it. Future research should also concentrate on utilizing immersive VR training applications, while additionally exploring the integration of large language models to augment interactive learning in mental health care. Finally, this review underscores the necessity in health education research involving VR to communicate research findings using agreed terms and classifications, with the aim of providing a clearer and more comprehensive understanding of the research.

## Conclusions

This systematic review investigated the effect of VR training interventions on knowledge, skills, and attitudes in the assessment and treatment of mental health disorders. The results suggest that VR training interventions can promote knowledge and skills acquisition. Further studies are needed to evaluate VR training interventions as a learning tool for mental health care providers. This review emphasizes the necessity to improve future study designs. Additionally, intervention studies of immersive VR applications are lacking in current research and should be a future area of focus.

### Supplementary Information

**Additional file 1: Table 2.** Effects of VR training in the included studies: Randomized controlled trials (RCTs) and non-randomized studies (NRSs).

## Data Availability

Detailed search strategies from each database is available in the DataverseNO repository, 10.18710/TI1E0O.

## Abbreviations

VR: Virtual Reality
CAVE: Cave Automatic Virtual Environment
RCT: Randomized Controlled Trial
NRS: Non-Randomized study
VSP: Virtual Standardized Patient
IPS: Interactive Patient Scenario
VP: Virtual Patient
PTSD: Post Traumatic Stress Disorder
SP: Standardized Patient
AI: Artificial intelligence
HINN: Inland Norway University of Applied Sciences
PhD: Doctor of Philosophy
