# Supplementary material for: The effectiveness of virtual reality training on knowledge, skills and attitudes of health care professionals and students in assessing and treating mental health disorders: a systematic review
Source: BMC Med Educ. 2024 May 1;24:480. doi: 10.1186/s12909-024-05423-0 (PMC11064237; doi:10.1186/s12909-024-05423-0)
Supplement: Supplementary file 1 — Additional file 1: Table 2. Effects of VR training in the included studies: Randomized controlled trials (RCTs) and non-randomized studies (NRSs). [file 12909_2024_5423_MOESM1_ESM.docx]

**Table 2**: Effects of VR training in the included studies: Randomized controlled trials (RCTs) and non-randomized studies (NRSs)

**Knowledge:**

| Pantziaras, 2015  NRS  Single group pretest-posttest | Intervention  (n=32) | | Effect size | Summary |
| --- | --- | --- | --- | --- |
|  | Mean | SD |  |  |
| Pretest  Posttest  Pre vs. post | 7.44  8.47  -1.03 | 0.31  1.65  1.33 | * | Training session with the VP has an effect on psychiatric knowledge. |
| *We found no measures of effect size or figures that could be used to calculate effect size in this study  P< .001 | | | | |

| Matsumara, 2018  NRS  Controlled before and after study  (n=79) | Intervention  (n=43) | | Control  (n= 36) | |  | |
| --- | --- | --- | --- | --- | --- | --- |
|  | Mean | SD | Mean | SD | Effect size | Summary |
| Preintervention  Postintervention | 8.17  18.08 | 3.39  4.35 | 8.42  15.51 | 4.03  4.32 | Cohen’s d: 0.59  95 % CI: 0.14 -1.04 | Training with virtual patients moderately improved medical students’ acquisition of psychiatric knowledge. |
| P value preintervention= 0.76  P value postintervention= 0.01 | | | | | | |

| Liu, 2021  NRS  Prospective cohort study | Intervention (n=149) | Comparator (n=150) | Effect size | Summary |
| --- | --- | --- | --- | --- |
| Recognizing depression  Recognizing schizophrenia | 145 (97.3 %)  133 (89.3) | 147 (98.0 %)  140 (93.3) | Cramer’s V: 0.02*  Cramer’s V: 0.07** | No effects of virtual simulation on changing students' knowledge of mental disorders. |
| *P=.69 **P=.21 | | | | |

**Skills:**

| Fleming,  2009  RCT | Intervention VR Simulation (n=51) | | | | Comparator No Training  (n=51) | | | | Effect size | Summary |
| --- | --- | --- | --- | --- | --- | --- | --- | --- | --- | --- |
|  | Pretest | | Posttest | | Pretest | | Posttest | |  |  |
|  | Mean | SD | Mean | SD | Mean | SD | Mean | SD |  |  |
| Screening  Total score  Intervention  Total score  Referral  Total score | 53.24  52.55  42.94 | 16.09  13.86  14.28 | 67.67  58.37  66.05 | 12.42  15.89  14.02 | 54.41  53.73  43.53 | 15.58  19.34  17.79 | 58.13  51.67  64.48 | 15.51  18.09  12.01 | Cohen’s d:  0.67*  95% CI: 0.27 -1.07  Cohen’s d:  0.39*  95% CI: 0.00 - 0.78  Cohen’s d:  0.12**  95% CI:  -0.26 - 0.50 | Significant positive effect on posttest alcohol screening and brief intervention skills and no effect on referral skills in intervention vs  comparator group. |
| *P< .05 **P= ns | | | | | | | | | | |

| Foster, 2015  RCT  Posttest only | Intervention  (n=34)  VP | Comparator  (n=33)  Video | Effect size | Summary |
| --- | --- | --- | --- | --- |
| Suicide thoughts %  Suicide plan %  Suicide means %  Past suicide attempts %  Family history of suicide % | 88.2  88.2  9.1  73.5  45.4 | 75.8  75.8  18.2  63.6  33.3 | Cramer’s V: 0.16*  Cramer’s V: 0.16*  Cramer’s V: 0.13*  Cramer’s V: 0.10*  Cramer’s V: 0.12* | No significant differences between the VP and video groups for suicide risk questions. |
| *P= ns | | | | |

| Satter, 2012  RCT  Posttest only | Intervention | | Intervention | | Comparator | | Effect size | 95 % CI | Summary |
| --- | --- | --- | --- | --- | --- | --- | --- | --- | --- |
|  | Fixed avatar  (n=10) | | Choice avatar (n=10) | | Text-based training  (n=10) | |  |  |  |
|  | Mean | SD | Mean | SD | Mean | SD |  |  |  |
| Diagnostic accuracy, major depressive disorder  Fixed avatar vs. Text:  Choice avatar vs. Text  Fixed avatar vs. Choice avatar | 5.10 | 1.52 | 5.00 | 1.76 | 2.80 | 1.40 | Cohen’s d: 1.57  Cohen’s d: 1.38  Cohen’s d: 0.06 | 0.57 - 2.57  0.40 - 2.35  -0.81 –  -0.93 | Avatar technology was better than traditional text-based methods in helping the participants to diagnose major depressive disorder and posttraumatic stress disorder. |
| Diagnostic accuracy, posttraumatic stress disorder  Fixed avatar vs. Text:  Choice avatar vs. Text  Fixed avatar vs. Choice avatar | 5.50 | 1.58 | 4.70 | 1.64 | 3.50 | 1.72 | Cohen’s d: 1.21  Cohen’s d: 0.71  Cohen’s d: 0.49 | 0.25 – 2.16  -0.18 – 1.61  -0.39 – 1.38 |  |

**Knowledge and skills:**

| Albright, 2018  RCT | VR training presimulation | | | VR training postsimulation | | | Effect size | Summary |
| --- | --- | --- | --- | --- | --- | --- | --- | --- |
|  | Mean | SD | Total | Mean | SD | Total |  |  |
| Within treatment Composite score  (n=117) | 2.82 | 0.79 | 117 | 3.40 | 0.89 | 117 | Cohen’s d_z_: 0.77* | Participants in the simulation group had significantly higher scores in the poststimulation survey than in the presimulation survey. |
| *The study did not provide confidence interval information  P < .001 | | | | | | | | |

**Attitudes:**

| Hitchcock, 2019  NRS  Single group pretest-posttest | Intervention group  (n=100) | | | Effect size: | | Summary |
| --- | --- | --- | --- | --- | --- | --- |
|  | Mean | SD | |  |  |  |
| Substance abuse attitude scale:  Presimulation  Postsimulation | 2.6  2.7 | .48  .55 | * | | No significant positive effect of simulation on attitudes. | |
| *We found no measures of effect size or figures that could be used to calculate effect size in this study.  P= ns | | | | | | |

**Perceived competence:**

| Hitchcock, 2019  NRS  Single group pretest-posttest | Intervention group  (n=100) | | | Effect size: | Summary |
| --- | --- | --- | --- | --- | --- |
|  | Mean | SD | |  |  |
| Perceived competence scale:  Presimulation  Postsimulation | 2.5  3.2 | .82  .46 | * | | The intervention improved perceived competence. |
| *We found no measures of effect size or figures that could be used to calculate effect size in this study.  P= .05 | | | | | |
